# The Prognostic Gender-Related Value of the Systemic Immune-Inflammation Index in Patients With Acute Coronary Syndrome

**DOI:** 10.31083/RCM44305

**Published:** 2026-01-23

**Authors:** Christos Kofos, Andreas S Papazoglou, Barbara Fyntanidou, Athanasios Samaras, Panagiotis Stachteas, Athina Nasoufidou, Aikaterini Apostolopoulou, Paschalis Karakasis, Alexandra Arvanitaki, Marios G Bantidos, Dimitrios V Moysidis, Nikolaos Stalikas, Dimitrios Patoulias, Apostolos Tzikas, George Kassimis, Nikolaos Fragakis, Efstratios Karagiannidis

**Affiliations:** ^1^Second Department of Cardiology, General Hospital ‘Hippokration', Aristotle University of Thessaloniki, 54642 Thessaloniki, Greece; ^2^Department of Cardiology, Athens Naval Hospital, 11521 Athens, Greece; ^3^Department of Emergency Medicine, AHEPA University Hospital, 54636 Thessaloniki, Greece; ^4^Medical School, Aristotle University of Thessaloniki, 54124 Thessaloniki, Greece; ^5^Cardiovascular Center Aalst AZORG Ziekenhuis, 9300 Aalst, Belgium; ^6^Second Propaedeutic Department of Internal Medicine, Faculty of Medicine, School of Health Sciences Aristotle, University of Thessaloniki, 54124 Thessaloniki, Greece

**Keywords:** acute coronary syndrome, systemic immune-inflammation index, SII, prognostic ratio, gender differences, all-cause mortality, inflammatory biomarker

## Abstract

**Background:**

Inflammation has recently been identified as a critical regulator of the pathophysiology and prognosis of acute coronary syndrome (ACS). The systemic immune–inflammation index (SII), derived from platelet, neutrophil, and lymphocyte counts, has gained attention as a potential marker for predicting adverse outcomes in cardiovascular diseases. However, the prognostic value of the SII, particularly in relation to gender differences, has not been extensively studied.

**Methods:**

Thus, we conducted a retrospective cohort study of 835 patients hospitalized for ACS at Hippokration Hospital, Thessaloniki, Greece, between 2017 and 2023. The SII was calculated using blood samples taken at admission. Logistic and Cox regression models were used to evaluate the relationship between the SII and all-cause mortality, with stratified analyses conducted according to gender. Receiver operating characteristic (ROC) analysis, Kaplan–Meier survival curves, and restricted cubic spline (RCS) modeling were also performed to assess the discriminative ability and non-linear associations of the SII with mortality.

**Results:**

A total of 835 patients were included, with a median follow-up of 25 months. An elevated SII was independently associated with increased long-term mortality, with patients in the highest SII quartile exhibiting a 2.3-fold higher risk of death compared to those in the lowest quartile (adjusted hazard ratio (aHR) = 2.31, 95% confidence interval (CI): 1.60–3.32; *p* < 0.001). The optimal cut-off value for the SII was identified as 1864.19. Gender-stratified analyses revealed a stronger prognostic value in women compared to men (area under the curve (AUC) = 0.70 vs 0.58; *p* = 0.018). The Kaplan–Meier and Cox regression analyses confirmed significantly worse survival for patients with SII levels above this threshold (*p* < 0.05). The RCS modeling demonstrated a non-linear relationship between the SII and mortality, with a marked increase in risk at higher levels of the SII, especially in women.

**Conclusions:**

The SII is a simple, easily accessible biomarker that independently predicts mortality in ACS patients, with notable gender-specific differences in the prognostic value of the SII. Nonetheless, incorporating SII into routine risk assessment could enhance risk stratification and improve personalized treatment strategies, particularly in settings with limited resources.

## 1. Introduction

Acute coronary syndrome (ACS) remains one of the leading causes of morbidity and mortality worldwide [1], despite significant advances in diagnostic tools and treatment [2]. Inflammation plays a key role in the pathogenesis of atherosclerosis and its complications [3], including atherosclerotic plaque rupture and subsequent thrombosis, the key mechanisms in developing ACS [4]. However, despite its well-established role, systemic inflammation is discussed less frequently in clinical practice compared to traditional cardiovascular risk factors, and many cardiologists report limited awareness or uncertainty regarding how to incorporate inflammatory biomarkers into routine risk stratification and management [5]. Several inflammatory biomarkers, such as high-sensitivity C-reactive protein (hs-CRP) and cytokines (interleukin (IL)-6, IL-8, tumor necrosis factor-alpha (TNF-α)), have been extensively studied, but their prognostic value remains under debate [6, 7].

In recent years, hematological markers of inflammation derived from simple blood tests have attracted scientific interest as they are easily measurable, cost-effective, and available in daily clinical practice. Among these, the systemic immune-inflammation index (SII) has emerged as a promising predictor of cardiovascular events [8]. The SII is calculated as (platelet count × neutrophil count)/lymphocyte count, incorporating three key cellular components of the inflammatory response, neutrophils, which promote endothelial dysfunction and atherosclerotic progression [9], lymphocytes, which participate in the regulation of the immune response [10] and platelets, which play a central role in both thrombosis and atherogenesis [11].

Initially, SII was clinically applied in oncology as a prognostic marker in patients with hepatocellular carcinoma [12]; however, recent studies have shown that elevated SII levels are associated with adverse outcomes in several cardiovascular diseases, such as stable coronary artery disease (CAD) and acute myocardial infarction (AMI) [13, 14]. In particular, SII has been associated with increased mortality and major adverse cardiovascular events (MACE) in patients with ACS undergoing percutaneous coronary intervention (PCI) [15].

In addition, emerging evidence suggests the potential for gender-related differences in both inflammatory responses and cardiovascular outcomes [16]. In particular, women with ACS tend to present more often with atypical symptoms [17], undergo less aggressive therapeutic approaches, and experience increased short- and long-term mortality compared to men [18]. At the same time, hormonal and immunological variations may influence inflammatory activity and affect the prognostic value of hematological markers [19]. However, the potential differential prognostic value of SII between men and women with ACS has not been well examined.

The present study aims to evaluate the predictive value of SII on the risk of all-cause mortality in patients with ACS, emphasizing the potential existence of gender-related differences. A deeper understanding of the relationship between systemic inflammation, gender, and cardiovascular outcomes may help to improve individualized treatment strategies and reduce cardiovascular mortality.

## 2. Methods

### 2.1 Study Population

This retrospective cohort study analyzed consecutive patients hospitalized for ACS in the Second Department of Cardiology at Hippokration Hospital of Thessaloniki, Greece, between 2017 and 2023. Patients aged 18 or older were included in the study if diagnosed with ACS; pregnant women were not included. ACS was defined according to the Fourth Universal Definition of Myocardial Infarction and European Society of Cardiology (ESC) guidelines. Subtypes included ST-elevation myocardial infarction (STEMI), non-ST-elevation myocardial infarction (NSTEMI), and unstable angina. Diagnosis was based on a combination of clinical presentation, electrocardiographic changes, and elevated cardiac troponin levels (for myocardial infarction). For unstable angina, patients had ischemic symptoms with or without electrocardiogram (ECG) changes but without troponin elevation. Patients with missing data required for the main analyses (SII calculation, mortality outcome, gender, or diabetes mellitus (DM) history) were excluded. Venous blood samples were obtained within the first 24 hours of hospital admission. Complete blood counts were analyzed using Sysmex XN-1000 hematology analyzer (Sysmex Corporation, Kobe, Japan), with both internal and external quality control performed daily. Patients with conditions known to significantly affect systemic inflammation, including recent infection, autoimmune disease, active malignancy, or hematologic disorders, were excluded from the analysis.

This study received ethical approval from the Ethics Committee of Hippokration Hospital of Thessaloniki. The study was conducted following the principles outlined in the Declaration of Helsinki [20]. As this represents a retrospective study, individual informed consent was not required.

### 2.2 Study Endpoint and Follow-up Procedures

The primary endpoint of this study was all-cause mortality during the follow-up period of approximately 2 years. Patient follow-up was conducted through electronic health record review, telephone interviews, and, where necessary, contact with primary care physicians or family members to verify survival status. Mortality data were cross-checked against hospital records and national death registries to ensure accuracy.

### 2.3 Statistical Analysis

Descriptive statistics were calculated for the entire cohort. Continuous variables were summarized using means ± standard deviations for normally distributed data or medians (with interquartile range, IQR) for non-normally distributed data, while categorical variables were summarized as frequencies and percentages.

The primary analysis was performed using Cox proportional hazards models, which incorporate follow-up time and provide hazard ratios for mortality risk. Logistic regression was additionally applied as a supplementary analysis to provide odds ratios at a fixed follow-up point. Logistic regression models were initially performed for the entire cohort to evaluate the association between the SII index and mortality, adjusting for several covariates, including demographic (age, gender), clinical (history of heart failure, hypertension, DM, dyslipidemia, chronic kidney disease, family history of CAD), and lifestyle factors (smoking status). Medication use, especially anti-inflammatory drugs, was evaluated; however, statin therapy was not significantly associated with outcomes in univariate analyses and was therefore not included in adjusted models. Data on colchicine use were not available. Multivariable models were adjusted for covariates selected based on established clinical relevance and univariate associations within the cohort. Variables known to influence outcomes in ACS (age, gender, DM, hypertension, heart failure, and renal function) were pre-specified for inclusion. Additional variables with *p* < 0.10 in univariate analysis were considered for adjustment. No automated stepwise selection procedures were used. Gender-stratified analyses were then performed by running separate logistic regression models for men and women. Interaction between SII and sex was formally tested in logistic regression models (both unadjusted and adjusted) using an SII-sex interaction term, and the corresponding *p*-value was reported. Post-hoc, gender-stratified Spearman correlations were computed between SII and prespecified clinical/laboratory variables (age, white blood cells count (WBC), neutrophils, lymphocytes, platelets, creatinine, glucose, left ventricular ejection fraction (LVEF)); false discovery rate was controlled using the Benjamini–Hochberg method, and adjusted *p*-values are reported.

Moreover, subgroup analyses were performed in patients with and without DM, stratified by gender, since DM is both common in ACS and closely linked to systemic inflammation, while gender is known to modulate immune responses. This allowed us to explore whether the prognostic value of SII differs across these clinically and biologically relevant dimensions.

A receiver operating characteristic (ROC) curve analysis was used to assess the discriminative ability of the SII index for predicting mortality. An area under the curve (AUC) was calculated for the entire cohort, as well as separately for men and women. The DeLong test was employed to compare AUCs between genders to determine whether the predictive accuracy of the SII index differed between male and female patients.

To further assess the SII’s robustness as a mortality predictor, we first performed a Kaplan-Meier survival analysis [21] using the optimal cut-off value identified by Youden’s Index. We then conducted a sensitivity analysis using alternative thresholds based on the 25th, 50th, and 75th percentiles of the SII distribution, dividing patients into four groups accordingly. Study participants were categorized into four different SII groups based on SII quartiles, and log-rank tests were used to compare survival curves for the entire cohort. Pairwise log-rank *p*-values were adjusted using the Holm method to account for multiple comparisons. Cox proportional hazards regression was performed with the lowest quartile (Q1) as the reference. The proportional hazards assumption was assessed with Schoenfeld residuals. To address multiplicity in hazard ratio estimates, simultaneous 95% confidence intervals and adjusted *p*-values were calculated using Dunnett’s single-step procedure, which is specifically designed for multiple comparisons against a common reference group. Additionally, Cox proportional hazards models were used to assess the impact of the SII index and other covariates on time to all-cause mortality both in the entire cohort and in gender-related subgroups. Effect modification by sex was evaluated by including an SII-sex interaction term in the Cox regression model, and the corresponding *p*-value for interaction was reported.

To explore potential non-linear associations between the continuum of the SII index and mortality, we applied restricted cubic spline (RCS) regression [22] within a Cox proportional hazards model. Using four knots selected based on statistical criteria, we assessed how mortality risk varied across different values of the SII index while adjusting for relevant clinical factors. For each model (overall, men, and women), both the overall association and the non-linear component were tested, and *p*-values for nonlinearity are reported. This approach allowed us to capture complex patterns that a linear model might overlook and to formally evaluate whether the association deviated from linearity. To reduce the influence of extreme outliers, we trimmed the distribution of SII at the 1st and 99th percentiles. This approach retained 98% of the study population while minimizing the leverage of rare extreme values that could otherwise distort the shape of the spline function. Restricted cubic splines were then fitted with 4 knots placed at the 5th, 35th, 65th, and 95th percentiles of the trimmed SII distribution.

All analyses were conducted using IBM SPSS Statistics, version 28.0 (IBM Corp., Armonk, NY, USA) and R, version 4.4.2 (R Foundation for Statistical Computing, Vienna, Austria). The results were presented with 95% confidence intervals (CIs) and *p*-values.

## 3. Results

In total, 835 patients with ACS [27.7% women, median age: 65 years (IQR: 56–75 years)] were included in the study. During a median follow-up of 25 months (IQR: 24–26 months), 155 (18.6%) patients died. The most common comorbidities in the study population were arterial hypertension (49.4%), DM (27.2%), dyslipidemia (25.6%), and family history of CAD (15.0%), while 7.2% of patients had a history of chronic kidney disease and 54.3% were active smokers (Table 1). The median SII index of the total cohort was 752.75 (IQR 472.02–1360.83).

**Table 1.** **Baseline characteristics of the study population overall and stratified by gender**.

Characteristic		Overall (n = 835)	Female (n = 225)	Male (n = 610)	*p*-value
Demographics & vitals					
	Age (years)	64 ± 13	69 ± 14	62 ± 12	<0.001
	Systolic BP (mmHg)	133 ± 25	135 ± 24	132 ± 25	0.064
	Heart rate (bpm)	79 ± 18	80 ± 18	79 ± 18	0.60
Comorbidities					
	Hypertension, n (%)	412 (49.4)	134 (60)	273 (45)	<0.001
	Diabetes mellitus, n (%)	227 (27.2)	69 (31)	147 (24)	0.054
	Dyslipidemia, n (%)	214 (25.6)	55 (24)	156 (26)	0.70
	Smoking, n (%)	454 (54.3)	60 (27)	325 (53)	<0.001
	Heart failure, n (%)	22 (2.6)	7 (3.1)	15 (2.5)	0.60
	Chronic kidney disease, n (%)	60 (7.2)	20 (8.9)	30 (4.9)	0.037
ACS subtype					
	STEMI, n (%)	317 (38)	67 (30)	249 (41)	0.003
	NSTEMI, n (%)	252 (30)	66 (29)	186 (30)	0.70
	Unstable angina, n (%)	266 (32)	92 (41)	175 (29)	<0.001
Laboratory values					
	Total cholesterol (mg/dL)	171 ± 44	172 ± 43	170 ± 45	0.60
	LDL (mg/dL)	96 ± 38	96 ± 37	97 ± 38	>0.9
	HDL (mg/dL)	40 ± 12	45 ± 13	39 ± 11	<0.001
	Triglycerides (mg/dL)	127 (95, 173)	125 (96, 160)	127 (95, 178)	0.30
	Creatinine (mg/dL)	1.17 ± 0.99	1.18 ± 1.18	1.17 ± 0.91	<0.001
	Hemoglobin (g/dL)	13.43 ± 1.78	12.28 ± 1.59	13.85 ± 1.65	<0.001
	WBC (10^9^/L)	9.9 ± 3.5	9.4 ± 3.4	10.1 ± 3.5	0.006
	Platelets (10^9^/L)	242 ± 70	256 ± 73	236 ± 68	<0.001
	Neutrophils (%)	68 ± 11	70 ± 11	68 ± 11	0.051
	SII index	752.75 (472.02, 1360.83)	842 (544, 1448)	726 (472, 1239)	0.014
Echocardiography					
	LVEF (%)	48 ± 10	48 ± 11	47 ± 10	0.70

Data are presented as mean ± standard deviation, median (interquartile range), or number (percentage), as appropriate. *p*-values correspond to comparisons between women and men. ACS, acute coronary syndrome; STEMI, ST-elevation myocardial infarction; NSTEMI, non-ST-elevation myocardial infarction; SII, systemic immune-inflammation index; LVEF, left ventricular ejection fraction; BP, blood pressure; LDL, low-density lipoprotein; HDL, high-density lipoprotein; WBC, white blood cells.

Univariate regression analysis demonstrated a significant association between the SII index and all-cause mortality (*p* < 0.001, OR = 1.01 (95% CI: 1.00–1.01)). Gender-stratified analyses revealed that this association was significant for both women (*p* = 0.005, OR = 1.01, 95% CI: 1.00–1.01) and men (*p* < 0.001, OR = 1.01, 95% CI: 1.00–1.01). Multivariate regression analyses, adjusted for clinically relevant covariates, confirmed these findings in the overall cohort (*p* < 0.001, adjusted OR (aOR) = 1.01, 95% CI: 1.00–1.01), as well as in the female cohort (*p* = 0.02, aOR = 1.01, 95% CI: 1.00–1.01), and the male cohort (*p* < 0.001, aOR = 1.01, 95% CI: 1.00–1.01) (**Supplementary Table 1**). However, in both unadjusted and adjusted models, the SII–sex interaction term was not statistically significant (*p*_interaction = 0.740 and 0.684, respectively), indicating that the strength of association did not differ between genders.

In sex-stratified analyses, SII correlated very strongly with neutrophils and platelets and inversely with lymphocytes in both men and women (*p* < 0.001), consistent with its definition. Beyond these components, SII showed modest but directionally consistent associations with higher glucose (men ρ = 0.26, q < 0.001; women ρ = 0.36, q < 0.001), higher creatinine (women ρ = 0.19, q < 0.001), and lower LVEF (men ρ = –0.20, q < 0.001; women ρ = –0.27, q < 0.001). These associations were generally stronger in women.

**Fig. 1.**
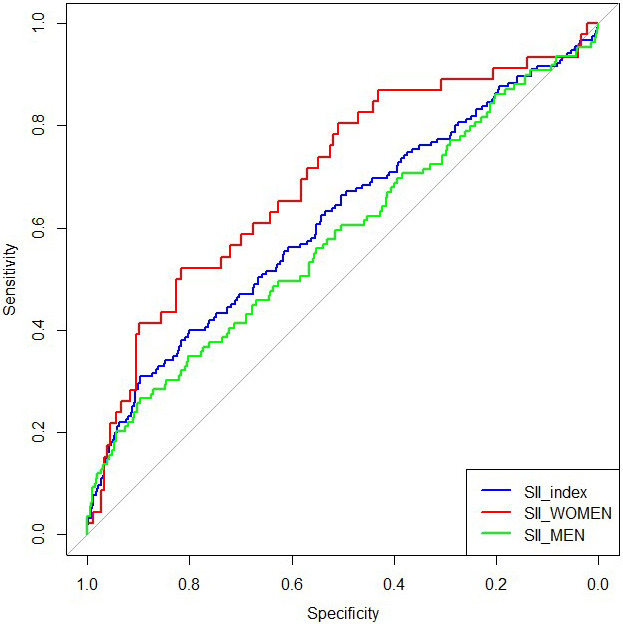
**Receiver operating characteristic (ROC) curve for all-cause mortality in the total study population, women, and men**. Systemic immune-inflammation index (SII) discriminated mortality risk with an area under the curve (AUC) of 0.70 (95% CI: 0.61–0.78) in women and 0.58 (95% CI: 0.52–0.64) in men. The difference between genders was statistically significant (*p* = 0.018, DeLong test; assumptions verified).

ROC curve analysis demonstrated an AUC of 0.60 (95% CI: 0.55–0.66) for the total population, using the optimal cut-off value of 1864.19, as calculated via Youden’s index, which corresponded to a sensitivity of 31% and a specificity of 90% (Fig. 1). Notably, gender-specific ROC analysis demonstrated an AUC of 0.70 (95% CI: 0.61–0.78) in women and 0.58 (95% CI: 0.52–0.64) in men (Fig. 1). The DeLong test confirmed a statistically significant difference in predictive accuracy between genders (*p* = 0.018).

A subgroup analysis was conducted to investigate the predictive performance of SII in patients with and without DM, stratified by sex. In the total population, the AUC for patients with DM was 0.68 (95% CI: 0.60–0.76), compared to 0.57 (95% CI: 0.50–0.64) in those without DM. Among women, the AUC was 0.65 (95% CI: 0.51–0.79) (n = 68) for those with DM and 0.72 (95% CI: 0.60–0.84) (n = 156) for those without. In contrast, men with DM had an AUC of 0.69 (95% CI: 0.59–0.79) (n = 152), while non-diabetic men had a lower AUC of 0.52 (95% CI: 0.44–0.66) (n = 459). These findings suggest notable variations in predictive performance depending on both DM status and sex (**Supplementary Table 2**).

**Fig. 2.**
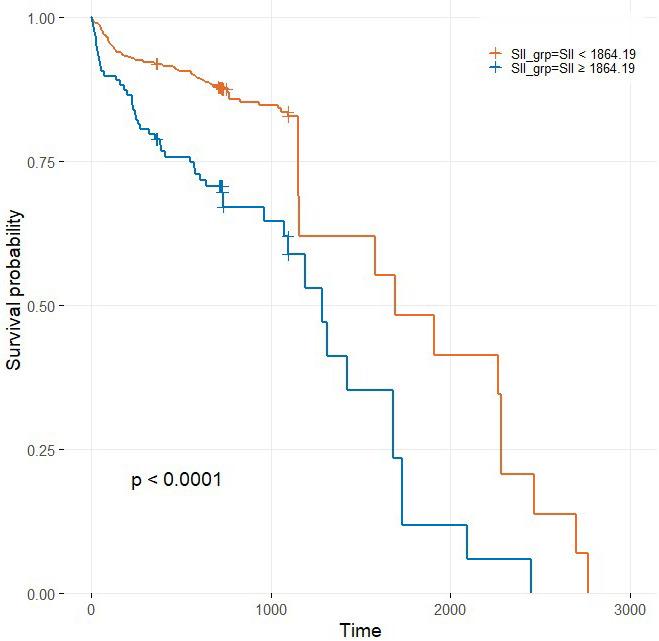
**Kaplan–Meier curves stratified by the optimal Youden cut-off of the systemic immune-inflammation index (SII): SII <1864.19 (red) vs SII ≥1864.19 (blue)**. Global log-rank *p* < 0.0001.

Cox proportional hazards regression analyses confirmed the prognostic significance of the SII index. Using the optimal cut-off value of 1864.19, determined by Youden’s Index, patients were categorized into two groups: those with a low SII (<1864.19) and those with a high SII (at or above this threshold). Survival analysis demonstrated significantly reduced survival probabilities in the high SII group (*p* < 0.0001) (Fig. 2). Furthermore, after adjusting for confounding variables (heart failure, hypertension, DM, dyslipidemia, family history of CAD, smoking, age, chronic kidney disease), patients with elevated SII exhibited a 2.31-fold increased risk of mortality (adjusted hazard ratio [aHR] = 2.31, 95% CI: 1.60–3.32, *p* < 0.001), underscoring the strong link between systemic inflammation and mortality risk (**Supplementary Table 3**). The SII-sex interaction term was statistically significant (*p* < 0.001), confirming that the association between SII and mortality differed between men and women.

**Fig. 3.**
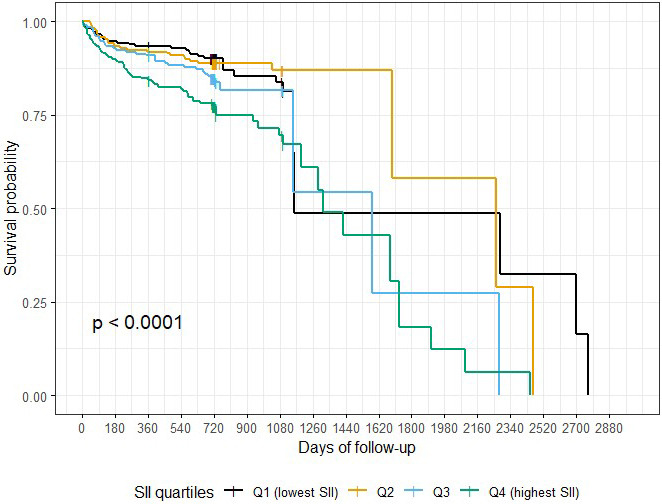
**Kaplan–Meier survival curves stratified by systemic immune-inflammation index (SII) quartiles (Q1–Q4)**. Global log-rank test *p* = 0.005; Holm-adjusted pairwise comparisons were significant for Q4 vs Q1 (*p* = 0.019) and Q4 vs Q2 (*p* = 0.042). The proportional hazards assumption was not violated (global Schoenfeld *p* = 0.40). In Cox regression, patients in Q4 had a significantly higher risk of mortality compared with Q1 (HR 2.15, 95% CI 1.19–3.88, *p* = 0.006, Dunnett-adjusted).

An additional analysis was conducted using SII quartiles (25th, 50th, and 75th percentiles) to explore the impact of alternative cut-off thresholds. The Kaplan-Meier curves demonstrated a graded decline in survival probability across increasing SII quartiles (*p* < 0.0001), supporting a dose-response relationship between SII levels and mortality risk (Fig. 3). The proportional hazards assumption was not violated (global Schoenfeld test *p* = 0.40). Pairwise log-rank comparisons with Holm adjustment confirmed significantly worse survival for Q4 compared with Q1 (*p* = 0.019) and Q4 compared with Q2 (*p* = 0.042), while other comparisons were not statistically significant. In Cox regression with Dunnett-adjusted simultaneous confidence intervals, Q4 was associated with more than double the risk of mortality versus Q1 (HR 2.15, 95% CI 1.19–3.88, *p* = 0.006). Q3 showed a borderline increase (HR 1.80, 95% CI 0.97–3.36, *p* = 0.067), while Q2 did not differ significantly from Q1 (HR 1.17, 95% CI 0.60–2.26, *p* = 0.896). When using Q1 as the reference, the risk of death increased across higher quartiles: HR 1.03 (95% CI: 0.83–2.73, *p* = 0.92) for Q2, HR 1.54 (95% CI: 0.54–2.57, *p* = 0.092) for Q3, and HR 2.40 (95% CI: 1.61–3.88, *p* < 0.001) for Q4. In the multivariate Cox regression analysis, patients in the highest SII quartile (Group 4) had a 2.43-fold higher risk of mortality compared to those in the lowest quartile (aHR = 2.43, 95% CI: 1.50–3.94, *p* < 0.001).

**Fig. 4.**
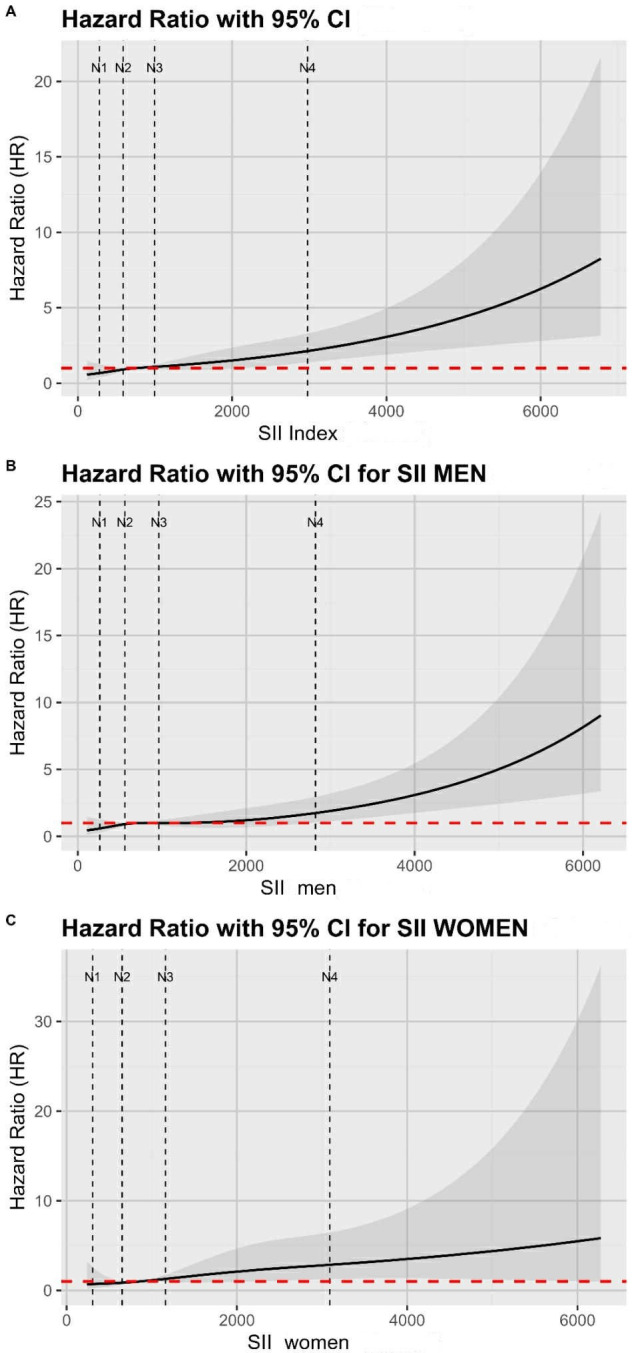
**Restricted cubic spline (RCS) Cox regression models of systemic immune-inflammation index (SII) and all-cause mortality after exclusion of extreme values (1st–99th percentiles)**. (A) Overall population, (B) men, and (C) women. Curves represent hazard ratios (solid line) with 95% confidence intervals (shaded area). The red dashed line indicates the reference (HR = 1). RCS were fitted with 4 node positions (knots) (vertical dotted lines) placed at the 5th, 35th, 65th, and 95th percentiles of the trimmed SII distribution. Node positions were: 279, 587, 997, and 2978 (overall); 262, 560, 964, and 2823 (men); and 305, 652, 1160, and 3091 (women).

The RCS analysis revealed a non-linear association (J-shaped pattern) between the SII Index and all-cause mortality after exclusion of extreme values (1st–99th percentiles) (overall *p* < 0.0001; *p* for nonlinearity = 0.0009). In the overall population, a significant increase in HR was observed starting at an SII value of 2122, beyond which the HR steadily rose, indicating a consistent elevation in mortality risk with higher SII values (Fig. 4A). This supports a non-linear dose-response relationship in the overall cohort. Among men, the spline curve remained flat in SII values up to 2770, beyond which the HR significantly increased (overall *p* < 0.0001; *p* for nonlinearity = 0.500), signaling a statistically significant increase in mortality risk (indicating that the association was important but approximately linear) (Fig. 4B). In contrast, the spline curve for women showed a different pattern (overall *p* = 0.015; *p* for nonlinearity = 0.026): a subtle increase in HR was evident at an SII value of 1410, but the association became significant when SII values exceeded 2265 and remained so until 5970, where the statistical significance seems to be lost (Fig. 4C). The revised Fig. 4 also includes vertical dashed lines to indicate the spline knots (N1–N4).

## 4. Discussion

The main finding of this study is the stronger predictive value of SII for long-term mortality in women with ACS compared to men, suggesting a potential sex-specific difference in the inflammatory response and its clinical implications. This aligns with prior evidence indicating that women may exhibit distinct immune activation patterns and inflammatory profiles, which could influence cardiovascular risk [23, 24]. Additionally, the study confirms that SII is an independent predictor of mortality in ACS overall, as elevated SII levels were significantly associated with increased long-term mortality even after adjusting for established cardiovascular risk factors. More specifically, in continuous analyses, the per-unit increase in SII yielded an OR of 1.01, reflecting the very small effect of a 1-unit change given the large scale of SII values. Over clinically meaningful increments, however, the risk estimates were more consistent with those from the categorical quartile analyses. Quartile analyses were therefore presented as they better reflect the non-linear, threshold-like association between SII and mortality and provide greater clinical interpretability. These findings reinforce the role of systemic inflammation in cardiovascular outcomes and support the clinical relevance of SII as a risk stratification tool.

A differential predictive performance of the SII index according to DM status and sex was demonstrated. One important thing to notice is that we focused our subgroup analysis on DM status stratified by gender, as both factors are strongly associated with systemic inflammation and cardiovascular outcomes. This approach provided clinically meaningful insights while avoiding excessive exploratory subgrouping. In the total cohort, patients with DM showed higher discriminative ability compared to non-diabetics, supporting the hypothesis that systemic inflammation may play a more prominent role in the pathophysiology of adverse events among diabetic patients [25]. Notably, men with DM demonstrated stronger predictive accuracy than their non-diabetic counterparts, whereas in women, the index performed slightly better in non-diabetic patients. These patterns suggest potential sex-specific and diabetes-related differences in how systemic inflammation influences cardiovascular risk, highlighting the need for further investigation. Such observations may inform a more personalized risk stratification in clinical practice.

These outcomes align with previous studies reporting a significant association between increasing SII and adverse cardiovascular events in patients with AMI and stable CAD [26]. Gao *et al*. [27] identified an optimal SII cut-off value of 713.9 × 10^9^/L (AUC: 0.709, 95% CI: 0.660–0.757) for predicting MACEs in ACS patients undergoing PCI. This cut-off demonstrated a fair predictive performance, with sensitivity of 63.6% and specificity of 71.2%. Although our overall AUC was slightly lower, our findings align closely, further validating the evidence supporting the prognostic value of the SII index.

A recent meta-analysis by Zhang *et al*. [15], further supports the prognostic value of SII in cardiovascular populations, showing a more than two-fold increased risk of MACEs in patients with elevated SII following PCI. More specifically, SII was linked to adverse outcomes, such as all-cause mortality, non-fatal AMI, and heart failure.

In their study, Shi *et al*. [28]. examined 744 ACS patients with coexisting chronic kidney disease and demonstrated that an elevated SII was independently associated with MACE. Similar to our approach, they employed Kaplan–Meier survival analysis and Cox regression modeling, further validating the prognostic significance of SII through time-to-event methods. Their ROC curve analysis identified an optimal cutoff of 1159.84 × 10^9^/L for predicting MACE, yielding an AUC of 0.706 (95% CI: 0.66–0.75), with SII outperforming other inflammatory indices such as neutrophil to lymphocyte ratio (NLR) and platelet to lymphocyte ratio (PLR). Our findings are in line with these results, as our ROC analysis also demonstrated solid predictive performance, especially in women, and our survival analysis confirmed the independent prognostic value of SII. Taken together, these complementary results highlight the consistency of SII’s prognostic utility across different ACS cohorts, including high-risk subgroups such as those with chronic kidney disease.

Sex differences seem to play a key role in inflammatory responses. Martínez de Toda *et al*. [29] showed that men tend to have higher levels of oxidative stress and inflammation, which might partly explain their shorter lifespan. Similarly, Trabace *et al*. [30] found that males are more prone to strong pro-inflammatory reactions in diseases like myocarditis, while females often show milder, more fibrotic responses. Adding to this, Wilkinson *et al*. [31] discussed how both sex chromosomes and hormones shape these immune differences, with females having stronger antiviral and vaccine responses but also a higher risk for autoimmunity. These findings highlight how important it is to consider sex in both research and treatment strategies. In cardiovascular disease specifically, prior studies have demonstrated that inflammatory biomarkers such as hs-CRP and IL-6 show potentially tighter associations with adverse outcomes in women compared to men [32], suggesting that systemic inflammation may be a particularly critical determinant of prognosis in females. This body of evidence provides a framework into which our findings on SII can be integrated, reinforcing the notion that sex-related immune differences are not limited to specific diseases but extend across cardiovascular conditions. Our observation that women exhibited increased risk at a lower SII threshold (1750) compared to men (2000) may reflect sex-related immunological differences. Estrogen has been shown to downregulate pro-inflammatory cytokines such as TNF-α and IL-6 while enhancing adaptive immune activity [33]. This hormonal modulation may lower the inflammatory threshold at which adverse outcomes manifest in women, consistent with the sex-specific prognostic patterns observed in our cohort. While we lack information on menopausal status or hormone replacement therapy, these factors are likely relevant given their influence on estrogen exposure, and future studies should incorporate them to clarify the underlying mechanisms.

Complementing these prior observations, our gender-stratified correlation analyses demonstrated that SII was more strongly linked to metabolic stress (glucose) and impaired cardiac function (LVEF) in women compared with men, beyond its expected relationships with neutrophils, lymphocytes, and platelets. In women, SII also showed modest associations with renal function (creatinine), again pointing to tighter biological coupling between systemic inflammation and important organ vulnerability. This pattern is consistent with evidence that women exhibit heightened inflammatory responses to metabolic disturbances [34] and are more prone to microvascular coronary dysfunction [35], both of which can amplify ischemic injury. Taken together, these correlations suggest that the prognostic strength of SII in women may arise not only from leukocyte–platelet interactions but also from the way inflammatory activity integrates with metabolic and myocardial stress pathways.

Beyond correlations, mechanistic pathways further support sex-specific differences in the prognostic value of SII. Neutrophils contribute to endothelial injury, oxidative stress, and thrombo-inflammation [36], and sex hormones have been shown to modulate neutrophil activation [37]. Platelets are central not only to thrombosis but also to inflammatory signaling through the release of cytokines and chemokines [38]; importantly, platelet reactivity has been reported to be higher in women, particularly under conditions of metabolic stress [39]. Also, estrogen has been shown, particularly at higher levels, to enhance lymphocyte survival and support regulatory T-cell activity [40]. Taken together, our findings indicate that in women, elevated SII may capture stronger inflammatory and thrombotic processes, consistent with the greater prognostic impact we observed at lower SII thresholds.

## 5. Limitations

Despite the strengths of our study, several limitations must be acknowledged. One notable limitation is its retrospective nature, which introduces the possibility of selection bias. Being conducted at a single institution also raises concerns about how well our results might apply to other populations or settings. While the sample size was sufficient for statistical evaluation, it may still fall short in terms of fully supporting some of our subgroup analyses, especially when determining ratio cut-offs or stratifying by biomarker levels. Medication use, especially anti-inflammatory drugs, was evaluated; however, statin therapy was not significantly associated with outcomes in univariate analyses and was therefore not included in adjusted models. Data on colchicine use were not available. Another limitation of our study is that SII was measured only once at baseline, whereas inflammatory activity may change over time. It is important to state that SII should be viewed not as a stand-alone predictor but as a simple, accessible biomarker that may complement established risk models. Information on menopausal status and hormone replacement therapy was not available in this cohort. Since estrogen plays a central role in modulating inflammatory pathways, this limits the mechanistic interpretation of our sex-specific findings and highlights an important area for future research. Furthermore, the AUC of 0.70 in women indicates only moderate discriminative ability. This limitation should be acknowledged; however, the consistent difference compared to men and the persistence of associations in adjusted analyses suggest that SII still provides clinically relevant prognostic information, particularly in women. Another significant limitation is that the ratio was only measured upon patients’ hospital admission for ACS. Therefore, a potential change in these inflammatory biomarkers over time and their predictive value could not be investigated. Future prospective, multi-center cohorts are warranted to confirm and further expand our observations.

## 6. Conclusions

This study highlights notable gender-specific differences in the prognostic value of SII, with a stronger association observed in women with ACS. These findings underscore the importance of further investigation into sex-adapted inflammatory risk stratification in cardiovascular disease. Given its simplicity, routine availability, and accessibility, SII may serve as a valuable biomarker for mortality risk assessment in ACS patients. Future research should explore the integration of SII into existing risk prediction models, alongside conventional clinical and biochemical markers.

## Availability of Data and Materials

Study data will be available upon reasonable request from the corresponding author (EK).

## Supplementary Material

Supplementary material associated with this article can be found, in the online version, at https://doi.org/10.31083/RCM44305.
