# Supplementary material for: The Prognostic Gender-Related Value of the Systemic Immune-Inflammation Index in Patients With Acute Coronary Syndrome
Source: Rev Cardiovasc Med. 2026 Jan 23;27(1):44305. doi: 10.31083/RCM44305 (PMC12873662; doi:10.31083/RCM44305)
Supplement: Supplementary file 1 [file 2153-8174-27-1-44305-s1.zip › Supplementary Material.docx]

Supplementary Table 1: Multivariate logistic regression for predictors of all-cause mortality in ACS patients

A. Total Population

| **Variable** | **Adjusted OR** | **95% CI** | **p-value** |
| --- | --- | --- | --- |
| **SII index** | 1.006 | 1.003–1.008 | <0.001 |
| **Heart failure** | 2.537 | 0.940–6.847 | 0.066 |
| **Hypertension** | 0.628 | 0.407–0.968 | 0.035 |
| **Diabetes mellitus** | 1.689 | 1.073–2.658 | 0.024 |
| **Dyslipidemia** | 0.562 | 0.338–0.933 | 0.026 |
| **Family history of CAD** | 0.574 | 0.281–1.173 | 0.128 |
| **Smoking** | 0.790 | 0.499–1.249 | 0.312 |
| **Age** | 1.029 | 1.011–1.047 | 0.002 |
| **CKD** | 1.901 | 0.992–3.642 | 0.053 |

B. Male subgroup

| **Variable** | **Adjusted OR** | **95% CI** | **p-value** |
| --- | --- | --- | --- |
| **SII** | 1.006 | 1.001–1.009 | <0.001 |
| **Heart failure** | 2.965 | 0.921–9.545 | 0.069 |
| **Hypertension** | 0.777 | 0.468–1.290 | 0.329 |
| **Diabetes mellitus** | 1.253 | 0.720–2.181 | 0.424 |
| **Dyslipidemia** | 0.639 | 0.354–1.153 | 0.137 |
| **Family history of CAD** | 0.565 | 0.252–1.266 | 0.166 |
| **Smoking** | 0.664 | 0.399–1.106 | 0.116 |
| **Age** | 1.012 | 0.990–1.034 | 0.301 |
| **CKD** | 2.233 | 1.000–4.985 | 0.050 |

C. Female subgroup

| **Variable** | **Adjusted OR** | **95% CI** | **p-value** |
| --- | --- | --- | --- |
| **SII** | 1.007 | 1.003–1.009 | 0.024 |
| **Heart failure** | 1.811 | 0.281–11.663 | 0.532 |
| **Hypertension** | 0.359 | 0.147–0.878 | 0.025 |
| **Diabetes mellitus** | 4.092 | 1.658–10.098 | 0.002 |
| **Dyslipidemia** | 0.326 | 0.112–0.952 | 0.040 |
| **Family history of CAD** | 0.491 | 0.094–2.572 | 0.400 |
| **Smoking** | 1.426 | 0.448–4.544 | 0.548 |
| **Age** | 1.082 | 1.040–1.125 | <0.001 |
| **CKD** | 1.354 | 0.415–4.411 | 0.615 |

Supplementary Table 2: Predictive performance of SII for all-cause mortality by diabetes status and gender

| **Subgroup** | **AUC** | **95% CI** |
| --- | --- | --- |
| **Total population** |  |  |
| With DM | 0.68 | 0.60–0.76 |
| Without DM | 0.57 | 0.50–0.64 |
| **Women** |  |  |
| With DM | 0.65 | 0.51–0.79 |
| Without DM | 0.72 | 0.60–0.84 |
| **Men** |  |  |
| With DM | 0.69 | 0.59–0.79 |
| Without DM | 0.52 | 0.44–0.66 |

Supplementary Table 3: Multivariate Cox Regression for predictors of all-cause mortality in ACS patients - total population

| **Variable** | **Adjusted HR** | **95% CI** | **p-value** |
| --- | --- | --- | --- |
| **SII Index (high vs. low)** | 2.307 | 1.596–3.317 | <0.001 |
| **Heart failure** | 2.079 | 1.006–4.300 | 0.048 |
| **Hypertension** | 0.833 | 0.576–1.204 | 0.330 |
| **Diabetes mellitus** | 1.264 | 0.860–1.856 | 0.233 |
| **Dyslipidemia** | 0.815 | 0.519–1.280 | 0.374 |
| **Family history of CAD** | 0.666 | 0.342–1.298 | 0.233 |
| **Smoking** | 1.052 | 0.690–1.602 | 0.814 |
| **Age** | 1.016 | 1.000–1.032 | 0.049 |
| **CKD** | 2.264 | 1.374–3.731 | 0.001 |
